# Health Literacy or Care Redesign in Atrial Fibrillation?

**DOI:** 10.1016/j.jacadv.2026.103092

**Published:** 2026-07-31

**Authors:** Paschalis Karakasis

**Affiliations:** Second Department of Cardiology, Aristotle University of Thessaloniki, General Hospital Hippokration, Greece

I read with great interest the systematic review and meta-analysis by Lathlean et al.^1^ The study is timely and clinically relevant. However, its central interpretive challenge is conceptual: the validity of the clinical inference depends on clearly distinguishing health literacy, health literacy interventions, and care redesign.

Health literacy refers to the patient’s ability to access, understand, appraise, and apply health information in ways that support informed decision-making and self-management.^1^ Health literacy interventions are therefore strategies designed to strengthen one or more of these capacities. However, such interventions do not map in a simple 1:1 fashion onto literacy itself. Educational videos, decision aids, reminder systems, telemonitoring tools, and clinician-facing software may all enhance understanding, but they also act through distinct behavioral, organizational, and therapeutic mechanisms. The distinction is especially relevant here because the review defines health literacy broadly and includes many interventions that extend well beyond education alone.

Many of the pooled interventions are therefore more accurately viewed not simply as literacy support, but as forms of care redesign. By care redesign, we mean structural modifications in atrial fibrillation (AF) management that alter how care is delivered, monitored, or operationalized, including integrated pathway-based care, clinician decision support, remote rhythm or blood pressure monitoring, reminder architectures, and facilitated follow-up. Several interventions included in the review incorporated precisely these elements. In that context, it is entirely plausible that at least part of the observed reduction in hospitalization and mortality reflects improved delivery of care rather than improved literacy per se.^2^, ^3^, ^4^

The internal pattern of results reinforces that concern. Knowledge improved substantially, but durability beyond 12 months was uncertain; self-efficacy effects were uncertain; and patient-action outcomes were mixed.^1^ If literacy is the proposed mediator, yet the downstream behavioral pathway remains inconsistent, then the clinical signal is unlikely to be explained by literacy alone. A more plausible interpretation is that education becomes clinically effective when embedded within multicomponent, pathway-driven AF care. That view is also consistent with prior AF integrated-care and shared decision-making data, where improvements in process quality have generally been more consistent than effects on downstream clinical behavior.^2^, ^3^, ^4^, ^5^

This is not merely semantic. It is a matter of construct validity. Pooling purely educational strategies together with multicomponent delivery-system interventions risks attributing benefit to the wrong mechanism. Future studies should distinguish literacy-targeted interventions from literacy-sensitive care and from true care-redesign strategies. Until then, we should be cautious not to mistake an enabling component for the principal driver of benefit.
